# Practical guidance on the initiation, titration, and switching of basal insulins: a narrative review for primary care

**DOI:** 10.1080/07853890.2021.1925148

**Published:** 2021-06-24

**Authors:** Roopa Mehta, Ronald Goldenberg, Daniel Katselnik, Louis Kuritzky

**Affiliations:** a National Institute of Medical Sciences and Nutrition Salvador Zubirán, Mexico City, Mexico; b LMC Diabetes & Endocrinology, Concord, Canada; c Diabetes and Metabolism Specialists, Shavano Park, USA; d University of Florida, Gainesville, USA

**Keywords:** Type 2 diabetes, basal insulin, primary care, initiation, titration, switching, overbasalization

## Abstract

Many patients with type 2 diabetes will ultimately require the inclusion of basal insulin in their treatment regimen. Since most people with type 2 diabetes are managed in the community, it is important that primary care providers understand and correctly manage the initiation and titration of basal insulins, and help patients to self-manage insulin injections. Newer, long-acting basal insulins provide greater stability and flexibility than older preparations and improved delivery systems. Basal insulin is usually initiated at a conservative dose of 10 units/day or 0.1–0.2 units/kg/day, then titrated thereafter over several weeks or months, based on patients’ self-measured fasting plasma glucose, to achieve an individualized target (usually 80–130 mg/dL). Through a shared decision-making process, confirmation of appropriate goals and titration methods should be established, including provisions for events that might alter scheduled titration (e.g. travel, dietary change, illness, hospitalization, etc.). Although switching between basal insulins is usually easily accomplished, pharmacokinetic and pharmacodynamic differences between formulations require clinicians to provide explicit guidance to patients. Basal insulin is effective long-term, but overbasalization (continuing to escalate dose without a meaningful reduction in fasting plasma glucose) should be avoided.

**Key messages**
- Primary care providers often initiate basal insulin for people with type 2 diabetes.
- Basal insulin is recommended to be initiated at 10 units/day or 0.1–0.2 units/kg/day, and doses must be titrated to agreed fasting plasma glucose goals, usually 80–130 mg/dL. A simple rule is to gradually increase the initial dose by 1 unit per day (NPH, insulin detemir, and glargine 100 units/mL) or 2–4 units once or twice per week (NPH, insulin detemir, glargine 100 and 300 units/mL, and degludec) until FPG levels remain consistently within the target range. If warranted, switching between basal insulins can be done using simple regimens.
- The dose of basal insulin should be increased as required up to approximately 0.5–1.0 units/kg/day in some cases. Overbasalization (continuing to escalate dose without a meaningful reduction in fasting plasma glucose) is not recommended; rather re-evaluation of individual therapy, including consideration of more concentrated basal insulin preparations and/or short-acting prandial insulin as well as other glucose-lowering therapies, is suggested.

## Introduction

Insulin is often necessary to attain glycemic targets in the long-term management of diabetes. Whereas people with type 1 diabetes tend to be under specialist care (usually led by an endocrinologist), >90% of patients with relatively uncomplicated type 2 diabetes (T2D) are managed by their primary care provider (PCP) [1]. Although preparations of basal insulin have been available since the 1940s, their daily use to normalize glycemic levels became standard in the 1970s [2]. In the last 20 years, the introduction of the long-acting basal insulin analogs glargine and detemir facilitated once-daily administration of basal insulin injections [3].

The American Diabetes Association (ADA) recommends that patients with T2D should initially receive oral glucose-lowering therapies to regulate their blood glucose, added to diet and lifestyle modifications [4]. Starting with metformin, further glucose-lowering treatments are added stepwise to maintain a target glycated hemoglobin (HbA_1c_) level (generally <7%); priority should be given to a glucagon-like peptide-1 receptor agonist (GLP-1RA) or sodium-glucose co-transporter-2 inhibitor (SGLT2i) with proven cardiovascular benefit if the patient has established atherosclerotic cardiovascular disease or is at high risk of developing cardiovascular disease, whereas an SGLT2i, with proven benefit on heart failure and/or chronic kidney disease, is preferred in patients with these comorbidities [4]. GLP-1RAs are generally recommended as the first injectable (although semaglutide is available in oral and parenteral formulations), because of multi-targeted effects that include lowering body weight [4], and in some cases reducing cardiovascular risk [5–8]. However, insulin should be the first injectable if there is evidence of ongoing catabolism (weight loss), symptoms of hyperglycemia (i.e. polyuria, polydipsia), very high levels of glycemia (HbA_1c_ >10% or fasting plasma glucose [FPG] ≥300 mg/dL), or if type 1 diabetes is likely [4]. Because T2D is progressive, many patients will eventually require daily insulin injections due to loss of pancreatic beta-cell function.

There is substantial clinical inertia among patients and healthcare providers regarding treatment intensification with insulin [9]. Reluctance to initiate basal insulin may stem from patient concerns about hypoglycemia, fear of injections, and a reluctance to accept (or lack of patient understanding of) the progressive nature of T2D; some PCPs may also lack experience in insulin initiation and have insufficient time to educate patients [10–15].

Insulin therapy is associated with a risk of hypoglycemia. First-generation (e.g. glargine 100 units/mL, detemir) and second-generation (e.g. degludec, glargine 300 units/mL) basal insulin analogs provide longer duration of action, more consistent plasma concentrations, and some clinical trials demonstrate a reduced risk of hypoglycemia, compared with intermediate-acting neutral protamine Hagedorn (NPH) insulin [16,17]. Newer and longer-acting second-generation analogs (insulin glargine 300 units/mL and insulin degludec) have been shown to lower the hypoglycemia risk compared to insulin glargine 100 units/mL [18,19]. A summary of key information on basal insulins is given in Table 1 [20].

**Table 1.** Summary of key information on basal insulins [20,25–29].

Basal insulin	Type	Approximate duration of effect (hours)	Risk of nocturnal hypoglycemia	Cardiovascular safety	Administration schedule
NPH	Intermediate-acting	<24	High	Not demonstrated	Once or twice daily with a syringe or pen
Detemir	Long-acting (first generation)	<24	Moderate	Not demonstrated	Once or twice daily by pen
Glargine 100 units/mL	Long-acting (first generation)	24	Moderate	Neutral	Once daily with a syringe or pen
Glargine 300 units/mL	Long-acting (second generation)	>30	Low	Not demonstrated	Once daily by pen
Degludec 100 or 200 units/mL	Long-acting (second generation)	≥42	Low	Neutral	Once daily with a syringe or pen

NPH: neutral protamine Hagedorn.

Patients receiving insulin are largely responsible for self-monitoring their blood glucose in order to guide self-titration. The role of PCPs (and Certified Diabetes Educators) is to educate patients on insulin administration and titration and make sure they know when to get in touch with the provider, based on predefined glucose readings. Nevertheless, unless clinicians make meaningful use of the data they receive, patients may lose interest in maintaining self-monitoring. A PCP should, therefore, help their patient understand when and why it is important to titrate therapy in a timely manner, and when other insulin formulations and/or glucose-lowering drugs are indicated [21]. For various reasons, some patients may benefit from a switch between basal insulin products or regimens, especially in light of newer preparations with more favourable efficacy and safety profiles [4].

In this review article, we provide practical guidance for PCPs on the initiation, titration, and switching of basal insulins.

## Initiating basal insulin

### Which patients are suitable for basal insulin?

The efficacy, safety, and convenience of basal insulins make them suitable for most patients with T2D who require treatment intensification in combination with most other classes of glucose-lowering agents. Despite this, there may be an increased risk of hypoglycemia when combining basal insulin with sulfonylurea; both agents are associated with hypoglycemic events, albeit that newer sulfonylureas (glipizide, glimepiride, and gliclazide) may carry a lower risk [22]. Thus, we generally recommend gradually tapering sulfonylurea once insulin is initiated. Discontinuation of concomitant thiazolidinedione is also suggested in patients at risk of heart failure [20].

**Figure 1.**
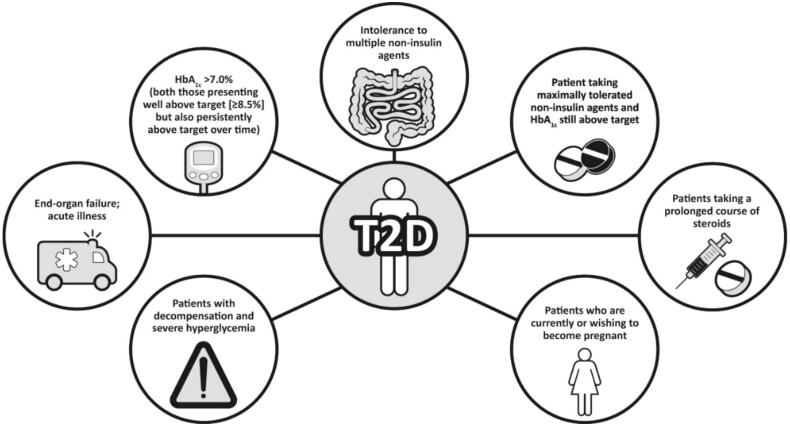
People with type 2 diabetes who are candidates for basal insulin. HbA_1c_: glycated hemoglobin; T2D: type 2 diabetes.

Given its greater affordability, patients who are well controlled on NPH may be able to continue on this therapy, since reductions in severe hypoglycemia with newer basal insulin analogs compared with NPH seen in some clinical trials may not be evident in clinical practice [23]. Although GLP-1RAs are usually recommended before insulin [4], for cost reasons, insulin is often the first injectable used. Common scenarios associated with patient suitability for insulin are shown in Figure 1.

Regardless of when basal insulin is initiated, it is important to explain to patients that insulin is an important add-on to their current treatment regimen and will help to further control their blood glucose. A common concern encountered in clinical practice is a patient with family members who deteriorated after starting insulin. Often this will have been because of long-standing hyperglycemia and clinical inertia earlier in their disease, but can erroneously be attributed to insulin. Other patients may feel a sense of shame or failure. It is important to explain to patients that insulin is needed because T2D is a progressive disease rather than because of anything they have or have not done regarding their existing glucose-lowering therapy [24].

Taking time in the consulting room to address patient concerns and show them the injection device and technique, as well as being available to address further questions and concerns after the consultation, is essential. A follow-up appointment or call to see how the patient is getting on will improve their confidence.

### Starting patients on basal insulin

Recommendations for initiating insulin analogs (glargine, degludec, or detemir) are summarized in Table 2 [25–29]. For patients with T2D, the starting dose is generally 10 units/day or 0.1–0.2 units/kg/day [4]. Although it is tempting to start patients on higher doses, especially if their body mass index is >35 kg/m^2^, starting with lower doses and ascertaining the patient’s responsiveness to insulin is prudent to avoid hypoglycemia [21].

**Table 2.** Summary of initiation, titration, and switching instructions for basal insulin analogs in patients with type 2 diabetes [25–29], and clinical recommendations from the authors.

Product	Description	Initiation	Titration*	Switching*
Basaglar^®^	Insulin glargine 100 U/mL in 3 mL prefilled KwikPen^®^ or Tempo Pen^TM^ delivery device	0.2 units/kg or up to 10 units once daily Adjustment of other glucose-lowering drugs may be required	Adjust the dose by 1 unit per day, or by 2–4 units once or twice per week, until FPG is 80–130 mg/dL (4.4–7.2 mmol/L) If FPG is >126 mg/dL (7 mmol/mol), suggest patient contacts PCP If hypoglycemia occurs (FPG <70 mg/dL [3.9 mmol/mol]), reduce the dose by 2–4 units or 10% of the total dose	From another insulin glargine 100 U/mL or once-daily NPH: continue same dose and timing of daily injection From once-daily insulin glargine 300 U/mL or twice-daily NPH: use 80% dose initially to reduce hypoglycemia risk From another intermediate or long-acting insulin: dose adjustment may be needed and shorter-acting insulins and doses of any other glucose-lowering drugs may need to be adjusted
Lantus^®^	Insulin glargine 100 U/mL in 10 mL multidose vial or 3 mL SoloStar^®^ prefilled pen	0.2 units/kg or up to 10 units once daily Adjustment of other glucose-lowering drugs may be required	Adjust the dose by 1 unit per day, or by 2–4 units once or twice per week, until FPG is 80–130 mg/dL (4.4–7.2 mmol/L) If FPG is >126 mg/dL (7 mmol/mol), suggest patient contacts PCP If hypoglycemia occurs (FPG <70 mg/dL [3.9 mmol/mol]), reduce the dose by 2–4 units or 10% of the total dose	From another once-daily insulin glargine 100 U/mL or once-daily NPH: continue same dose and timing of daily injection From once-daily insulin glargine 300 U/mL or twice-daily NPH: use 80% dose initially to reduce hypoglycemia risk From another intermediate or long-acting insulin: dose adjustment may be needed and shorter-acting insulins and doses of any other glucose-lowering drugs may need to be adjusted
Toujeo^®^	Insulin glargine 300 U/mL in 1.5/3 mL SoloStar^®^ prefilled pen	0.2 units/kg up to 10 units once daily Adjustment of other glucose-lowering drugs may be required	To minimize the risk of hypoglycemia, titrate the dose no more frequently than every 3 to 4 d until FPG is 80–130 mg/dL (4.4–7.2 mmol/L) If FPG is >126 mg/dL (7 mmol/mol), suggest patient contacts PCP If hypoglycemia occurs (FPG <70 mg/dL [3.9 mmol/mol]), reduce the dose by 2–4 units or 10% of the total dose	From another once-daily long- or intermediate-acting insulin: continue same dose and timing of daily injection but expect a higher dose will be needed if converting from insulin glargine 100 U/mL From twice-daily NPH: use 80% dose initially to reduce hypoglycemia risk Monitor glucose and titrate dose frequently in the first weeks of therapy, and adjust the dose of other glucose-lowering therapies per standard of care
Levemir^®^	Insulin detemir 100 U/mL in 3 mL FlexTouch^®^ or 10 mL multidose vial	10 units or 0.1–0.2 units/kg given once daily in the evening or divided into a twice-daily regimen	Adjust the dose by 2 units every 3–7 d until FPG is 80–130 mg/dL (4.4–7.2 mmol/L) If FPG is >126 mg/dL (7 mmol/mol), suggest patient contacts PCP If hypoglycemia occurs (FPG <70 mg/dL [3.9 mmol/mol]), reduce the dose by 2–4 units or 10% of the total dose	Conversion from insulin glargine can be done on a unit-to-unit basis, but some patients may require higher Levemir doses than NPH Monitor glucose and titrate dose frequently in the first weeks of therapy, and adjust the dose of other glucose-lowering therapies per standard of care
Tresiba^®^	Insulin degludec 100 or 200 U/mL in 3 mL FlexTouch^®^ prefilled pen, or 10 mL multidose vial	10 units once daily	To minimize the risk of hypoglycemia, titrate the dose no more frequently than every 3 to 4 d until FPG is 80–130 mg/dL (4.4–7.2 mmol/L) If FPG is >126 mg/dL (7 mmol/mol), suggest patient contacts PCP If hypoglycemia occurs (FPG <70 mg/dL [3.9 mmol/mol]), reduce the dose by 2–4 units or 10% of the total dose	Start Tresiba at the same unit dose as the total daily long- or intermediate-acting insulin unit dose

FPG: fasting plasma glucose; NPH: neutral protamine Hagedorn; PCP: primary care provider; U: units.

*Based on the authors’ clinical practice and recommendations (not all information is included in the product’s prescribing information).

Patients should understand that their initial dose is lower than that which will eventually be required and will increase, usually up to 35–45 units/day (approximately 0.5 units/kg/day), but potentially higher in some cases [21]. They should be instructed to inject basal insulin at the same time each day. The newer basal insulins offer flexibility in this regard: insulin degludec allows for intervals of 8–40 h between injections without compromising glycemic control or safety [30], and insulin glargine 300 units/mL can be injected up to 3 h on either side of the usual dosing time [31]. Patients should be taught to recognize the signs of hypoglycemia, such as shaking, sweating, dizziness, and weakness, as well as how to manage hypoglycemic episodes. Minor episodes can be resolved using glucose tablets, or a sugary snack or drink [32]. A practical suggestion is to advise patients to take 15 g of carbohydrates, wait for 15 min and re-test, repeating the process if levels remain <70 mg/dL [21,33]. Nasal glucagon has recently become available [34] and is useful because it can be administered if consciousness has been impaired.

**Figure 2.**
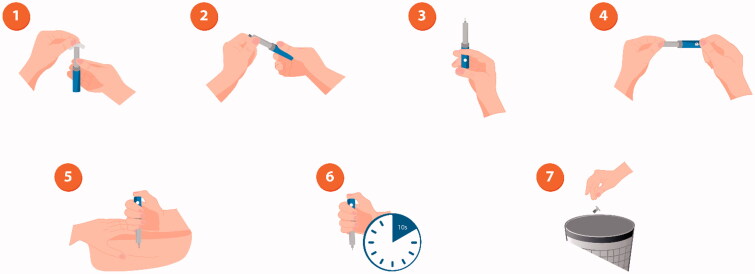
Illustration of correct injection technique with an insulin pen. 1: Remove cap and disinfect the top of the pen with an alcohol wipe; 2. Remove the paper tab from the needle and screw the needle tightly onto the pen top; 3. Remove air bubbles by dialling two units of insulin, holding the pen with the needle pointing upwards, and pressing the dose button. A drop of insulin should be visible at the top of the needle. If not, repeat the process until a drop appears; 4. Turn the dial so that the number of units you need is shown in the dose window; 5. Hold the pen with the needle pointing straight down towards the injection area and push the needle into the skin, then press the dose button; 6. Hold the pen in place for approximately 10 seconds to make sure all the insulin has been injected, and then remove the needle from the skin; 7. Put the plastic cap on the needle, unscrew it from the pen and throw the needle away in a sharps bin. Put the cap back on the pen.

It is vital to teach patients the importance of correct injection technique [35]; many patients, even those who are confident in injecting, still do this incorrectly [36]. Correct administration encompasses determining the correct dose, consistently administering into the subcutaneous space, and avoiding intramuscular injection and needle sticks (Figure 2). For patients using a vial and syringe, the correct dose must be chosen (patients often confuse doses due to different types of syringe), drawn up correctly, and the full dose must be injected before withdrawing the needle. For pens and syringes, injections should be rotated around sites on the limbs and abdomen/torso to prevent injection-site pain, irritation, and/or hypertrophy [4].

A sample patient checklist for starting basal insulin is provided in Table 3.

**Table 3.** Patient checklist for initiating and titrating basal insulin.

Checklist item	✓
Patient understands the reasons for starting basal insulin and agrees to this	
Demonstration of the syringe/pen, how to inject correctly – ideally the patient should demonstrate they can do this	
Patient understands the need to rotate the site of injection	
Patient understands the dose, frequency (once or twice daily), and need to inject at the same time each day, as far as possible	
Patient aware of storage and transport requirements for their insulin, and best before date*	
Patient has a blood glucose meter and is able to demonstrate they know how to use it correctly	
Patient understands what is meant by hypoglycemia, what the signs of hypoglycemia are, and actions to take depending on the severity	
Explanation provided regarding the need to initiate at a lower dose to avoid hypoglycemia and titrate upwards over time	
Patient understands their fasting plasma glucose target and how to adjust the dose accordingly	
Plan for titration agreed between primary care provider and patient, including what to do in the event of high readings	
Other medications, including glucose-lowering medications, review and adjust as appropriate	
Follow-up schedule agreed between primary care provider and patient	

*Normally 4–6 weeks from the date of first use or 8 weeks for degludec.

## Titrating basal insulins

### Why is dose titration necessary?

Active dose titration of basal insulin is important both for maintaining glycemic control and preventing hypoglycemia, and instructing patients in self-titration based on self-monitoring of FPG improves glycemic control [4]. Despite this, many patients who start on the standard 10 units/day or 0.1–0.2 units/kg/day of basal insulin often do not receive adequate instructions on titrating their dose [21]. It is important for patients to understand at the outset that the basal insulin dose will likely need to be increased incrementally, determined by daily self-measurement of FPG and that achieving the optimal dose may take several weeks or months.

Patient-led titration of basal insulin has been shown to be similarly effective to physician-led titration but requires education and support from PCPs, and tools to help patients self-adjust their dose as needed [37]. To avoid patients becoming discouraged during the process, it may help them to know that typical doses of basal insulin vary between patients and over time, but are commonly in the 35–45 units/day (approximately 0.5 units/kg/day) range or higher in our experience, and may be as high as 50–100 units/day (up to 1.0 units/kg/day) in some patients. This is primarily influenced by body weight and thus, conversely, some patient groups (e.g. Asian patients) will rarely need doses higher than 20 units/day.

### Practical recommendations on insulin titration

Simple algorithms for dose titration of insulin analogs have been suggested, acknowledging the need for individual FPG targets based on patient characteristics and instructions from individual product labels (Table 2) [4,25–29]. A simple rule is to gradually increase the initial dose by 1 unit per day (insulin detemir, and glargine 100 units/mL) or 2–4 units once or twice per week (insulin detemir, glargine 100 and 300 units/mL, and degludec) until FPG levels remain consistently within the target range (we suggest the ADA target of 80–130 mg/dL [4.4–7.2 mmol/L] but published recommendations vary) [4,20,21,38–42]. Patients could be advised to monitor FPG and titrate daily, or take consecutive FPG measurements on three consecutive days during the week and take the average or the lowest reading to guide titration [20,21]. If FPG is consistently high (e.g., >126 mg/dL), the patient should dose-adjust according to guidance, or ask their PCP for advice on how to proceed [4,25–29].

If hypoglycemia occurs (FPG <70 mg/dL [3.9 mmol/L], level 1 hypoglycemia defined by the ADA) [42], the patient should reduce the basal insulin dose by 2–4 units or 10% of the total dose; the PCP should follow-up with the patient soon afterward to reinforce education on preventing and recognizing hypoglycemia. If repeated episodes occur (1–2 per week), the titration schedule should be revisited [20].

Doses of other glucose-lowering medications may need to be adjusted as the insulin dose is increased [20]. Metformin, GLP-1RAs, and SGLT2is are usually continued, whereas thiazolidinedione is generally stopped when a patient starts on basal insulin [20]. Sulfonylurea is generally tapered as the insulin dose increases, in order to reduce hypoglycemia risk. However, discontinuing sulfonylurea may require an increase of up to 20 units of basal insulin to compensate [20]. Dosing insulin at bedtime and sulfonylurea in the day is a widely accepted regimen that can help to reduce the risk of hypoglycemia [43].

PCPs should keep in close contact with their patient at the start of titration (initially every few days and then weekly or biweekly) to receive FPG readings, answer questions, and ensure titration is proceeding as agreed [20]. After 3 months, HbA_1c_ can be measured, and titration should continue. If the HbA_1c_ target is not attained once FPG goals have been achieved, the remaining “culprit” excess glucose load must, by default, be postprandial. Appropriate steps to correct for excess postprandial glucose include rapid-acting insulin, glinides (i.e. repaglinide, nateglinide), and GLP-1RAs. Recent meta-analyses that have compared GLP-1RAs to rapid-acting insulin in patients with maximized basal insulin indicate that, while both treatments are similarly effective, GLP-1RAs are associated with less hypoglycemia and weight loss versus weight gain with rapid-acting insulin [44,45].

### Adjusting basal insulin in specific scenarios

Advice regarding missed or double doses depends on the pharmacologic characteristics of each basal insulin product (Table 1) [20,25–29]. In general, if the patient thinks they have missed a dose, they should test their FPG and contact their care team. Again, the flexibility and stable glucose-lowering action of long-acting insulin analogs help in this regard. For insulin degludec, for example, patients who realize they have missed a dose can inject it during waking hours the same day, as long as they ensure at least 8 h between consecutive injections [27]. If a double dose is taken, we suggest that patients should test their blood sugar frequently during the day, eat a snack and, in the night, wake up every 2–3 h to test glucose (with an extra snack if the reading is <126 mg/dL).

Changes in physical activity, meal patterns, renal or hepatic function, or acute illness may require greater dose adjustments that should only be made under medical supervision [25–29]. In general, patients may need to temporarily increase their basal insulin doses when unwell. If hospitalized, doses may also have to be modified and often titrated back to pre- hospitalization doses afterward [20]. Reduction and gradual elimination of insulin in patients who required temporary insulin, because of hospitalization or surgery, for example, may sometimes be needed. In the case of a patient suffering acute illness with reduced intake of food and fluids, the usual dose of basal insulin should be continued but with more frequent testing of FPG [20].

Any procedure that requires fasting will necessitate insulin adjustment. This is often the case with minor medical and surgical examinations or procedures, such as colonoscopy. On the day before the examination or procedure, patients should be advised to test their FPG four times (before each main meal and at bedtime) and to replace solids with fluids (sugary drinks) with the goal of ingesting 15–30 g of carbohydrates every 1–2 h [46]. If hypoglycemia occurs, the patient should take glucose tablets. Basal insulin should be taken at the usual dose. If dosing twice daily, the evening dose should be reduced to 80% if the evening FPG reading is <180 mg/dL, if the patient has frequent nocturnal hypoglycemia, has a usual FPG in the 72 mg/dL range, or had low blood glucose earlier in the day. Metformin, dipeptidyl peptidase-4 inhibitors, and thiazolidinedione should be taken as normal the day before the procedure, but GLP-1RAs, SGLT2is, sulfonylurea, and meglitinide should be omitted. On the day of the examination or procedure, the patient should be advised to omit other glucose-lowering drugs and take a half dose of basal insulin, take their insulin and glucose meter to the clinic, and aim for an FPG of 145–216 mg/dL. After the procedure, the usual insulin dose can be taken at bedtime and other glucose-lowering agents should not be taken that day. Once the patient is eating again, they can resume the usual doses of basal insulin and other agents [46].

Another common scenario is what to do when traveling internationally or across time zones. Firstly, the patient may require a letter from their PCP to allow them to take their injectable medication through airport security. Most intermediate- and long-acting basal insulins can be stored without refrigeration, according to the instructions for use (up to 28 d for glargine 100 units/mL, but 56 d for degludec and glargine 300 units/mL once at room temperature or opened for use) [25,27,28]. Assuming a change in the time zone of a few hours, patients on once-daily regimens may simply be able to take their daily dose at the usual time on the day of and after travel or may need to adjust the timing of their dose around travel to ensure adequate coverage. For twice-daily regimens, time zone changes of a few hours one way or the other may not necessitate any changes, but medium- and long-haul trips will lead to significant lengthening or shortening of the travel day, and potentially unusual mealtimes, which will require more frequent monitoring and adjustments to the timings of daily doses (Table 4) [27,29,32].

**Table 4.** Guidance on managing basal insulin for patients traveling internationally or across time zones.

Type of trip	Guidance
Short-haul trips (change in time zone of a few hours one way or the other)	Patients on once-daily regimens with longer-acting basal insulins that provide a consistent level of insulin for more than 24 h after a dose (e.g. 42 h for degludec) [27] may simply be able to take their daily dose at the usual time on the day of and after travel. Patients on once-daily regimens with basal insulins, such as detemir, that only have clinical activity lasting ∼24 h [29] may need to adjust the timing of their dose around travel to ensure adequate coverage.
	For patients on twice-daily regimens, changes may not be necessary.
Medium- and long-haul trips	Patients on twice-daily regimens will be affected by significant lengthening or shortening of the travel day and potentially unusual mealtimes.
Traveling east (short day)	When traveling east (short day), it is suggested to take the usual morning dose of a twice-daily basal insulin schedule, but to take the evening dose earlier than usual during travel [32]. After arrival, the patient may need to reduce or skip the next morning’s dose in the new time zone (depending on FPG reading) to account for overlap with the previous evening’s dose. In the case of missing the morning dose, the evening dose may be taken earlier than usual before resuming the normal schedule on the next day [32].
Traveling west (long day)	The patient may be advised to take a higher evening dose of their twice-daily regimen. They should not take additional insulin when they arrive at their destination but should check their blood glucose before bed in the new time zone, and again in the morning, to ensure levels are not too low. They can then resume their usual schedule [32].

FPG: fasting plasma glucose.

## Switching between basal insulins

### Why switch between basal insulins?

**Figure 3.**
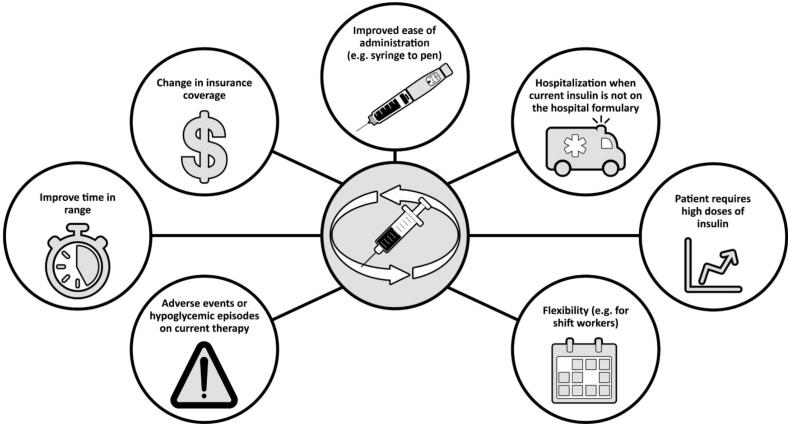
Reasons for switching between basal insulins.

There are various reasons for switching between basal insulins, both medical (e.g. adverse events or hypoglycemic episodes on current therapy) and practical (e.g. change of product availability or insurance coverage, or improved ease of use with newer delivery systems [pens vs. syringes]) (Figure 3). Recently, it has been shown that patients at risk of hypoglycemia had improved time in therapeutic range with insulin degludec 100 units/mL compared to glargine 100 units/mL [47]. Furthermore, patients whose weight-based insulin doses are sufficiently large that the volume of injection causes substantial discomfort (e.g. >80 units/day) may want to switch to a basal insulin of larger unit/volume (e.g. glargine 300 units/mL, degludec 200 units/mL) [48].

### How to switch between basal insulins

To minimize the risk of hyperglycemia/hypoglycemia, monitor blood glucose frequently in the first weeks of the new therapy and titrate the dose of insulin and other glucose-lowering therapies per standard of care [4,20]. When switching, it is important to educate the patient that destabilization of their FPG may occur initially and that consequently monitoring frequency should be increased for a time. Communication with the healthcare team needs to be enhanced, and higher-risk activities (driving, sports, and some occupations) may necessitate special precautions for a period after the switch.

Recommendations for switching between basal insulins are given in Table 2 [25–29]. If switching between different insulin glargine 100 units/mL preparations, or between insulin glargine 100 units/mL and insulin degludec 100 or 200 units/mL, the starting dose should be the same as the dose of the product to be discontinued [25–27]. For patients switching from insulin glargine 100 units/mL to insulin glargine 300 units/mL, start at the same dose but expect a higher daily dose will be needed to maintain the same level of glycemic control; when switching from glargine 300 units/mL to 100 units/mL, use 80% of the 300 unit/mL dose [25,26,28]. If switching from insulin glargine to insulin detemir, the dose should be the same total daily dose, unit-for-unit (1:1) [29]. This is also generally the case when switching from insulin detemir to another intermediate-acting or long-acting insulin, although dose adjustment may be needed if switching from detemir to glargine 100 units/mL [25,26].

When switching from twice-daily NPH to basal insulin analog, the dosage should be started at 80% of the total NPH dose being discontinued to reduce hypoglycemia risk. If switching from once-daily NPH to glargine, the product labels generally suggest a unit-for-unit (1:1) conversion [25–29].

## Beyond basal insulin

Basal insulin is effective for glycemic control, but the progressive nature of T2D means that further measures will generally need to be taken. Initially, this involves increasing the basal insulin dose, but there is a need to avoid overbasalization (i.e. titrating to high levels when other options for glycemic control are indicated), which increases the risk of hypoglycemia [21]. Of course, before the question of further medications or actions is considered, it is important to make sure that basal insulin has been incrementally titrated to the appropriate target level in a timely manner. If HbA_1c_ remains above target despite adequately titrated basal insulin and FPG being at target, the ADA recommends that clinicians should re-evaluate individual therapy [4]. This is particularly recommended if the difference between bedtime and morning or postprandial and preprandial glucose is high (e.g. ≥50 mg/dL for bedtime:morning differential), in the case of hypoglycemia (whether the patient is aware of it or not), in patients with high variability in FPG, and/or if the basal insulin dose is greater than approximately 0.5–1.0 units/kg/day [4,22]. Problematic nocturnal hypoglycemia (often occurring in the setting of pregnancy, steroid therapy, or liver disease) could also be a reason to consider whether continuing to increase the basal insulin dose is warranted or whether other therapeutic approaches are needed.

Evaluation of whether and to what extent these or other issues are preventing the achievement of glycemic goals should prompt an increase in contact between the patient and their care team, even if the patient seems otherwise capable of largely self-managing their therapy and lifestyle. It may be that some individuals who are not obviously struggling with managing their diabetes would in fact benefit from educational refreshers and further support regarding certain aspects. Simply increasing the insulin dose without further investigation is considered not likely to achieve the desired results in patients who are persistently not at target.

Rather than increasing the basal insulin dose beyond the suggested ceiling, other glucose-lowering agents should be added to basal insulin in a stepwise manner (if the patient is not already receiving them), depending on the need for weight loss, risk of hypoglycemia, and cost [4,22]. A GLP-1RA or SGLT2i with proven cardiovascular benefit should be added in the case of established cardiovascular disease or high cardiovascular risk (basal insulin is considered neutral with respect to cardiovascular events [49]), whereas an SGLT2i is preferred if chronic kidney disease or heart failure predominates [4,50]. Fixed-ratio combination injections of basal insulin plus a GLP-1RA (insulin glargine 100 units/mL plus lixisenatide [iGlarLixi], and insulin degludec plus liraglutide [IDegLira]) [51,52] are also available. Fixed-ratio combination therapy reduces the number of injections, can decrease HbA_1c_ more than either medication alone [53,54], and potentially offsets the weight gain associated with basal insulin with the weight reduction effects of GLP-1RAs. There is also the potential for less nausea than with a GLP-1RA alone [53], and the risk of severe hypoglycemia was not increased in clinical trials [51,52].

If the patient is still not at the HbA_1c_ target, additional doses of short-acting insulin with meals (basal-plus or basal-bolus insulin regimen) and use of premixed insulin (which combines short- and intermediate/long-acting insulin) can be considered [4]. Insulin degludec/insulin aspart (IDegAsp) was the first soluble combination of two insulin analogs [55], and is often best injected during the largest meal of the day. Patients may benefit from the use of concentrated insulin formulations in the case of obesity or insulin resistance [48].

## Conclusion

Basal insulin is often required in the management of T2D. Correct initiation, titration, and where appropriate, switching between preparations can facilitate good control of FPG. Patients should be encouraged to self-manage insulin injections and blood glucose monitoring. PCPs and patients need to work together to titrate the insulin dose over time to meet an agreed individual glycemic target. Patients should understand why and how they need to adjust their dose, managing situations like medical interventions, sick days, and travel. Newer long-acting basal insulins provide greater stability and easier administration than older preparations, with a lower risk of hypoglycemia. The transition between basal insulins can be done by following simple switching regimens. It is also important to understand the limits of basal insulin efficacy and that further treatment options are preferable to overbasalization.

## Supplementary Material

Supplemental Material
