# Supplementary material for: Practical guidance on the initiation, titration, and switching of basal insulins: a narrative review for primary care
Source: Ann Med. 2021 Jun 24;53(1):999–1010. doi: 10.1080/07853890.2021.1925148 (PMC8231382; doi:10.1080/07853890.2021.1925148)
Supplement: Supplemental Material [file IANN_A_1925148_SM1019.pdf]

Initiate

10 units/day or
0.1–0.2 units/kg/day

Help patient understand the need for basal insulin, and reasons for starting conservatively

Teach correct **injection technique**, how to self-monitor blood glucose, and to recognize and deal with **hypoglycemia**

Keep in **close contact** with patient during initial period after treatment start

Titrate

Individualized FPG target (ADA recommends 80–130 mg/dL*)

Share decision-making and confirm appropriate glycemic goals with patient

Educate patient on **titration methods** and events that might alter scheduled titration

Adjust other glucose-lowering medications and consider discontinuing SU if relevant

Hypoglycemia: reduce dose by 2–4 units/10% of total

Switch

E.g., because of adverse events, hypoglycemic episodes, change of product availability or insurance coverage, improved ease of use/reduction of injection volume

Not all basal insulins are the same: know the appropriate doses when switching from one to another

Generally same dose and timing when switching between once-daily basal insulins (glargine 100U [and 100U to 300U], detemir 100U, degludec 100/200U, once-daily NPH)

Start at 80% dose when switching:

- from glargine 300U to glargine 100U
- from twice-daily NPH to glargine 100U, detemir 100U, or degludec 100/200U

Be aware of overbasalization

I.e., continuing to escalate dose without meaningful reduction in FPG†

Consider instead adding‡

- GLP-1RA (consider fixed-ratio combinations)
- Oral glucose-lowering drug(s)
- Prandial/premixed insulin
- Concentrated insulin (reduced injection volume in obesity/ insulin resistance)

*Guidance varies and some recommendations suggest stricter control
†Generally, do not escalate basal insulin beyond doses >0.5 U/kg/day
‡Options are not necessarily in order of preference and depend on individual patient factors
ADA, American Diabetes Association; FPG, fasting plasma glucose; GLP-1RA, glucose-like peptide-1 receptor agonist
